# Recent Advances in Nanomedicine for the Diagnosis and Therapy of Liver Fibrosis

**DOI:** 10.3390/nano10101945

**Published:** 2020-09-29

**Authors:** Xue Bai, Gaoxing Su, Shumei Zhai

**Affiliations:** 1 School of Chemistry and Chemical Engineering, Shandong University, Jinan 250100, China; baixue1013@163.com; 2 School of Public Health, Cheeloo College of Medicine, Shandong University, Jinan 250012, China; 3 School of Pharmacy, Nantong University, Nantong 226001, China

**Keywords:** nanomedicine, liver fibrosis, diagnosis, therapy, theranostics, targeted drug delivery

## Abstract

Liver fibrosis, a reversible pathological process of inflammation and fiber deposition caused by chronic liver injury and can cause severe health complications, including liver failure, liver cirrhosis, and liver cancer. Traditional diagnostic methods and drug-based therapy have several limitations, such as lack of precision and inadequate therapeutic efficiency. As a medical application of nanotechnology, nanomedicine exhibits great potential for liver fibrosis diagnosis and therapy. Nanomedicine enhances imaging contrast and improves tissue penetration and cellular internalization; it simultaneously achieves targeted drug delivery, combined therapy, as well as diagnosis and therapy (i.e., theranostics). In this review, recent designs and development efforts of nanomedicine systems for the diagnosis, therapy, and theranostics of liver fibrosis are introduced. Relative to traditional methods, these nanomedicine systems generally demonstrate significant improvement in liver fibrosis treatment. Perspectives and challenges related to these nanomedicine systems translated from laboratory to clinical use are also discussed.

## 1. Introduction

Liver fibrosis is an important pathological and repair process in chronic liver disease, which is caused by chronic viral hepatitis, alcohol, and non-alcoholic steatohepatitis (NASH), and autoimmune liver disease [1,2,3]. It has been reported that liver diseases caused 4.6% of all deaths in the Asia-Pacific region, 2.7% in the USA, and 2.1% in Europe in 2015. The Asia-Pacific region holds more than half of the global population and accounted for 62.6% of all deaths due to liver diseases globally in 2015. Chronic hepatitis B virus (HBV) infection caused more than half of the deaths due to cirrhosis and other chronic liver diseases, followed by alcohol consumption, non-alcoholic fatty liver disease, and chronic infection with hepatitis C virus [4]. With persistent damage, live fibrosis develops into cirrhosis and, even to hepatocellular carcinoma, together with a series of complications, including hepatic encephalopathy, hepatic failure, and portal hypertension [5,6].

Liver fibrosis is currently diagnosed based on ultrasound imaging and blood testing, both of which lack precision [7]. Chemical drugs [8], Chinese herbal medicines [9], and monoclonal antibodies [10] are also being developed for the treatment of liver fibrosis. These approaches aim to remove injurious stimuli, suppress hepatic inflammation, down regulate hepatic stellate cell (HSC) activation, and promote matrix degradation [11]. However, these approaches exhibit limited therapeutic efficiency and have side effects. Therapeutic methods with enhanced therapeutic efficiency and targeted capabilities need to be developed. Precise diagnostic methods are also needed to monitor the progression of the disease.

Nanomedicine involves the design and application of nanoparticles (NPs) in the diagnosis and treatment of diseases [12,13,14]. As an important area of nanotechnology research, nanomedicine has greatly contributed to biomedicine in recent decades. Finely designed nanostructures have been fabricated as effective therapeutic agents for liver fibrosis with specific site-targeting abilities [15,16,17]. Nanostructures have also been developed as nanoagents for contrast enhancement or nanoprobes for the diagnosis of liver fibrosis [18]. Numerous inorganic or organic NPs have thus far been extensively investigated for the diagnosis and treatment of liver fibrosis, including metal oxide NPs [18], metal NPs [19], lipid NPs [20], polymer NPs [21], and protein NPs [22]. The various composition, controllable shape, size, and modifiable surface properties of NPs provide to them superior advantages, including controlled drug release, cell-tissue gap penetration, high contrast, prolonged duration in the bloodstream, improvement of the pharmacokinetics of drugs, and reduction of toxic side effects [23]. A greater significance of such systems is that they allow the integration of diagnosis and therapy in one nanoplatform [24].

The current review summarizes potential targets and the application of emerging nanomedicine systems for liver fibrosis diagnosis and therapy, including liposomes, polymer NPs, protein NPs, inorganic NPs, and hybrid NPs. Major research gaps, challenges, and coping strategies for the treatment of liver fibrosis by using nanomedicine are also discussed.

## 2. Potential Targets of Liver Fibrosis

**Figure 1:**
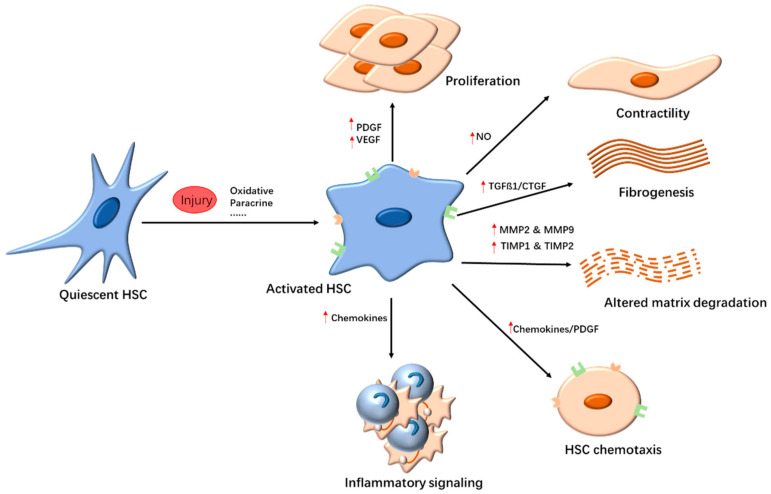
Hepatic stellate cell (HSC) activation. The pathways of HSC activation include initiation and perpetuation stages. Initiation is stimulated by reactive oxygen species (ROS), paracrine stimuli, and so on. The continuous stimulation could induce HSCs into myofibroblast cells, and the perpetuation phase occurs, which is involved in the change of HSC behavior, including proliferation, contractility, fibrogenesis, altered matrix degradation, chemotaxis, and inflammatory signaling. Abbreviations: CTGF, connective tissue growth factor; HSC, hepatic stellate cell; MMP, matrix metalloproteinase; NO, nitric oxide; PDGF, platelet-derived growth factor; TGF-β1, transforming growth factor β1; TIMP, tissue inhibitor of metalloproteinase; VEGF, vascular endothelial growth factor.

Activated HSCs are involved in the inflammatory response, fibrogenesis, and angiogenesis in liver fibrosis (Figure 1). They are at the center of liver fibrosis. Therefore, HSC-targeted strategies can be developed for the treatment of liver fibrosis. Alternative strategies include anti-inflammatory agents and inhibition of collagen deposition.

### 2.1. Targeting HSCs

Comprising about 13% of total liver cells, HSCs exist in the sinus space and come in direct contact with hepatic epithelial cells and endothelial cells [25]. In their normal state, HSCs are quiescent and mainly participate in vitamin A (VA) metabolism and fat storage. If the liver suffers from injuries, HSCs are activated and transformed into myofibroblasts. Activation of HSCs is a hallmark of liver fibrosis. Activated HSCs typically express smooth muscle actin (α-SMA); in addition, they synthesize and secrete the extracellular matrix (ECM). ECM deposition changes the structure and function of liver tissue, which is the root cause of liver fibrosis [26,27]. Therefore, activated HSCs are among the important targets for liver fibrosis therapy. Numerous signaling molecules are involved in the activation of HSCs, with TGF-β and PDGF being the important ones [27,28,29]. Therefore, blocking the TGF-β or PDGF signaling pathways is an effective strategy for the treatment of liver fibrosis. Protease inhibitors, such as camostat mesilate (FOY305), can neutralize TGF-β [30]. As a multi-target receptor tyrosine kinase inhibitor, sorafenib, targets the Raf/ERK signaling pathways and PDGF receptor and can effectively attenuate experimental liver fibrosis, inflammation, and angiogenesis [31]. Moreover, many anti-fibrosis drugs, such as the semisynthetic analog of fumagillin-TNP-470 [32] and the fungal metabolite-OPC-15161 [33], suppress the activation and proliferation of HSCs. Moreover, ROS contributes to liver fibrosis by promoting the activation and proliferation of fibroblasts and myofibroblasts, as well as the activating the TGF-β pathway in an autocrine manner [34]. Antioxidants, such as vitamin E [35], silymarin [36], N-acetylcysteine [8], resveratrol [37], quercetin [9], phosphatidylcholine [38], and glutathione [39], can inhibit the activation of HSCs and reduce liver fibrosis. These drugs benefit patients with alcoholic liver disease and NASH.

HSCs are also involved in hepatic angiogenesis and hepatic sinus vascular remodeling. When stimulated by inflammatory factors or hypoxia, HSCs can directly express VEGF and angiopoietin-1, influencing angiogenesis in the liver [40,41]. Pathological angiogenesis is related to the process of liver fibrosis and cirrhosis [42]. During liver fibrosis, fibrous scar tissue presses against the portal vein and central vein, leading to increased intrahepatic resistance. Simultaneously, liver sinus capillarization and fibrous scar obstruction also increase the resistance of blood flow and oxygen diffusion. These processes result in low oxygen conditions in the liver and gene expression, which are sensitive to oxygen concentration such as hypoxia-inducible factors (HIFs). Pathological angiogenesis cannot improve the oxygen level in the liver because of the high permeability of new blood vessels induced by VEGF. Therefore, pathologic angiogenesis and hypoxemia interfere with normal tissue repair and promote the development of liver fibrosis [43]. Pathological angiogenesis plays an important role in liver fibrosis and thus has been considered as an important therapeutic target for the reversal of liver fibrosis. One study found that the knockout of HIF-1α in rats significantly ameliorated liver fibrosis, indicating that improving intrahepatic hypoxia could effectively treat such a disease [44].

### 2.2. Anti-Inflammatory Response

An inflammatory response to autocrine or paracrine stimulation prompts HSC activation and proliferation. HSC activation consists of two stages: initiation and perpetuation [25,27]. Early changes in gene expression and phenotype represent the initiation stage. Initiation is mainly induced by cytokines, or other stimuli from cells around HSCs and act as paracrine pathways [27]. Reactive oxygen species (ROS) released from Kupffer cells can directly stimulate and activate HSCs [45]. By cascade amplification of inflammatory response, a large number of inflammatory cells infiltrate the damaged sites, and secrete inflammatory cytokines, leading to HSC activation and proliferation [46]. Continuous stimulation can induce HSCs into myofibroblast cells, inducing the perpetuation stage. The activated HSCs subsequently release chemokines, further aggravating the inflammatory response. In this stage, HSC promotes inflammation, fibrosis, and cell proliferation in the autocrine and/or paracrine pathway. Therefore, the inflammation response plays an important role in liver fibrosis. Anti-inflammatory drugs such as corticosteroids [47], colchicine [48], and ursodeoxycholic acid [49] have been used to treat liver fibrosis. Another anti-inflammatory strategy involves the application of specific receptor antagonists to neutralize inflammatory cytokines. In one study, the antifibrotic and anti-inflammatory effects of IL-10 were observed in patients infected with hepatitis C [50]. Moreover, hepatic macrophages participate in the pathogenesis of liver fibrosis by the secretion of inflammatory factors. Targeting hepatic macrophages is also an effective anti-inflammatory technique for the treatment of liver fibrosis [51].

### 2.3. Inhibition of Collagen Deposition

The main clinical feature of liver fibrosis is the excessive deposition of ECM, particularly collagen. Collagen I is the main component in ECM, and the cross-linking of collagen I is significantly increased in liver fibrosis. Therefore, collagen I reduction has been adopted to treat liver fibrosis. In one study, the monoclonal antibody AB0023, which is an inhibitor of the matrix remodeling enzyme LOXL2, inhibited liver fibrosis by regulating the cross-linking of collagen I [52]. In another study, the human monoclonal antibody GS-6624 was used for the treatment of NASH-induced liver fibrosis [11].

## 3. Nanomedicine in Liver Fibrosis Diagnosis

**Figure 2:**
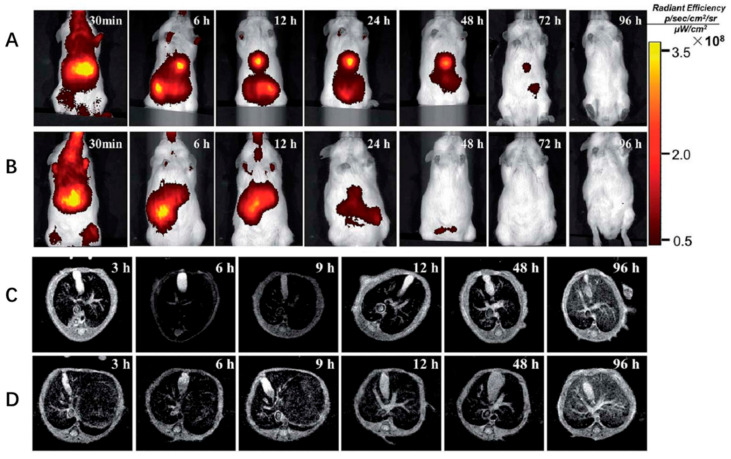
In vivo optical imaging and MRI of liver fibrosis using SPIO@SiO_2_–ICG–RGD. (**A**,**C**) A model of hepatic fibrosis in mice. (**B**,**D**) Healthy mouse model (control). Adapted with permission from [18]. Copyright RSC publishing, 2018.

For liver diseases, early detection of liver fibrosis would be helpful for treatment. Unfortunately, most cases of liver disease are diagnosed late because of no symptoms. Current strategies for the diagnosis of liver fibrosis rely on an invasive biopsy which would cause damage for the patients [53]. Recently, magnetic resonance imaging (MRI) has been developed as a method with high diagnostic accuracy for the detection of fibrosis [54]. Magnetic NPs play an important role in the diagnosis and imaging of liver fibrosis [55]. For example, dextran stabilized superparamagnetic iron oxide NPs (D-SPIONs) with high blood compatibility and low cytotoxicity was used as an MRI contrast agent for liver fibrosis detection [56]. D-SPIONs enhanced image contrast of tissue and led to a 55% decrease in the pixel intensity, and therefore improved the contrast difference between the fibrotic tissue and the rest of the extracellular matrix rich hepatic parenchyma at the fibrosis stage significantly. Citrate-coated ultrasmall iron oxide NPs were also shown to provide a good MRI of liver fibrosis [57]. In one study, Fe_3_O_4_ NPs coated with SiO_2_ and then coupled with indocyanine green (ICG) and arginine–glycine–aspartic acid (SPIO@SiO_2_–ICG–RGD) were constructed for HSC targeting and early detection of liver fibrosis (Figure 2) [18]. Fe_3_O_4_ NPs and ICG as the photographic developers for T_2_ MRI and near-infrared (NIR) imaging, respectively. NIR fluorescence (NIR) and MRI revealed that SPIO@SiO_2_–ICG–RGD could elicit accurate identification of fibrotic regions in the liver. These NIR hybrid NPs combined imaging and MRI and provided higher sensitivity and spatial resolution for liver fibrosis detection, compared with MRI alone.

Furthermore, zero-valent iron (ZVI)-based NPs were also fabricated as novel contrast agents for MRI. After functionalized with liver specific polysaccharide pullulan and fluorescent carbon dots, a dual imaging contrast agent (P@ZVI-Cdts) was obtained. The efficiency of the developed systems for targeted liver imaging and optical imaging has been successfully demonstrated in vivo. The high r1 relaxivity enables ZVI NPs to be a competent T_1_ MRI contrast agent for various clinical applications including diagnosis of liver fibrosis [58].

In addition to MRI, the combination of an ultrasound agent with a targeting peptide has been reported for the early and non-invasive diagnosis of liver fibrosis. One study found that core–shell perfluorooctyl bromide (PFOB) coated with poly(lactic-co-glycolic acid) (PLGA) polymers and modified with a cyclic RGD (cRGD–PLGA–PFOB NPs) exhibited powerful ultrasound molecular imaging features, including high-contrast imaging among liver fibrotic stages and adjacent tissues [59].

## 4. Nanomedicine for Liver Fibrosis Therapy

### 4.1. NPs as Therapeutic Agents

**Figure 3:**
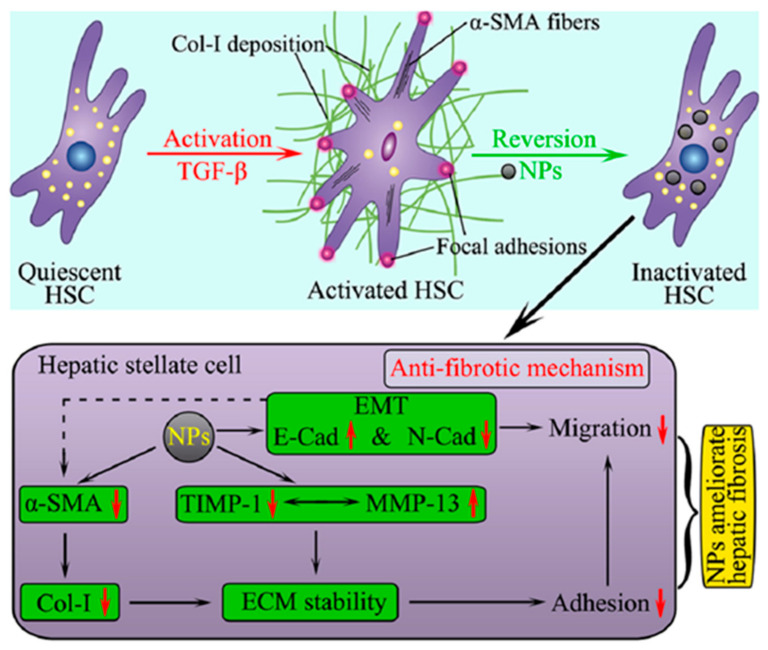
Model for TiO_2_ NPs and SiO_2_ NPs ameliorated fibrosis, adhesion and migration of HSCs. TiO_2_ NPs and SiO_2_ NPs can suppress the expression of α-SMA and deposition of Col-I induced by TGF-β. ECM was degraded by upregulating MMP-13 and downregulating TIMP-1. Therefore, adhesion of LX-2 cells was reduced. Furthermore, NPs stimulated the expression of E-Cad and reduced the expression of N-Cad, and, therefore, aggravated the migratory phenotype. Reproduced with permission from [63]. Copyright American Chemical Society, 2018.

Owing to their distinctive bioactive properties, inorganic NPs alone can be used as therapeutic agents for liver fibrosis therapy [60,61,62]. Both titanium dioxide NPs (TiO_2_ NPs) and silicon dioxide NPs (SiO_2_ NPs) can inhibit the expression of collagen I and α-SMA. They also facilitate collagen I degradation by upregulating matrix metalloproteinases (MMPs) and downregulating tissue inhibitors of metalloproteinases (TIMPs), indicating the potential antifibrotic activities of TiO_2_ NPs and SiO_2_ NPs in vitro [63]. These NPs further exhibit anti-adhesive and anti-migratory effects by regulating epithelial−mesenchymal transition (EMT) gene expression and revert TGF-β-activated HSCs to a quiescent state (Figure 3). Owing to their anti-inflammatory properties, cerium oxide NPs reduce liver steatosis, portal hypertension, and liver fibrosis in rats [64]. Oral exposure of citrate-functionalized Mn_3_O_4_ NPs can protect the liver from carbon tetrachloride (CCl_4_)-induced cirrhosis, fibrosis, and oxidative stress because of the increased antioxidant properties of Mn_3_O_4_ NPs upon acid treatment in the stomach [65]. ZnO NPs also ameliorate liver fibrosis by reducing lipid peroxidation, oxidative stress, and inflammation in dimethylnitrosamine-induced liver damage [66].

In addition to metal oxide NPs, other types of inorganic NPs are also used to treat liver fibrosis. A study found that gold NPs reduced liver fibrosis in a rat model of ethanol- and methamphetamine-induced liver injury by inhibiting the activity of Kupffer cells and HSCs [67]. The mechanism involves the regulation of AKT/PI3K and MAPK signaling pathways by gold NPs, thereby reducing pro-inflammatory cytokine secretion and oxidative stress. Another study reported that vitamin E-modified selenium NPs can attenuate liver fibrosis by reducing oxidative stress [68].

### 4.2. NPs as Drug Carriers without Targeting Ligand for the Treatment of Liver Fibrosis

The liver is the main metabolic and excretory organ in the body in which NPs can accumulate and accomplish passive targeting because of their size. Thus, NPs have been widely used as drug carriers for the treatment of liver fibrosis. Lipid-based NPs have been recognized as the most powerful vehicles because of their good biocompatibility and low toxicity (Table 1) [69]. CCAAT/enhancer-binding protein alpha (CEBPA), a master transcriptional factor in the liver, resets the natural gene regulatory mechanism of hepatocytes to reduce fibrosis and reverse liver dysfunction. Small activating RNA oligonucleotide therapy (CEBPA-51) formulated in liposome NPs can upregulate CEBPA, thereby reducing fibrosis [70]. After Cur-mNLCs treatment, pro-inflammatory cytokines, collagen fibers and α-SMA were reduced, while hepatocyte growth factors (HGF) and MMP2 were increased. Cationic lipid NPs loaded with small interfering RNA to the procollagen α1(I) gene (LNP-siCol1α1) can be retained in the liver of fibrotic mice and accumulate in nonparenchymal liver cells, specifically blocking procollagen α1(I) expression and inhibiting liver fibrosis progression without noticeable side effects [20].

Polymer-based NPs have been fabricated as drug carriers for the treatment of liver fibrosis (Table 1). In one study, ketal cross-linked cationic nanohydrogel particles were synthesized to deliver Cy5-labeled anti-col1α1 siRNA, which enhanced carrier and payload accumulation in the fibrotic tissue and prevented fibrosis progression [73]. PLGA and eudragit have also been employed as drug carriers. In one study, phyllanthin was carried by PLGA to reduce liver marker enzymes, namely alanine aminotransferase and aspartate aminotransferase, as well as collagen [74]. In another study, silymarin was delivered by eudragit NPs for the treatment of liver fibrosis by decreasing the expression of TNF-α, TGF-β1, TIMP-1, and CK-19. Moreover, nanoformulations were also found to increase HGF and MMP-2 expression and the MMP-2/TIMP-1 ratio [76].

In addition, inorganic NPs such as silica-based NPs were prepared as drug carriers because of their porous structures (Table 1). Salvianolic acid B (SAB) loaded rhodamine B covalently grafted mesoporous silica NPs (SAB@MSNs-RhB) were prepared for liver fibrosis therapy through the [77]. The SAB@MSNs-RhB formulation exhibited improved cellular uptake, sustained drug release, and enhanced efficacy in anti-ROS/hepatic fibrosis. Small interfering tenascin-C was delivered by mesoporous silica NPs to reduce the expression of TnC, an ECM glycoprotein, consequently reducing the secretion of inflammatory cytokines and hepatocyte migration [78]. Compared with hesperetin alone, hesperetin loaded on PEGylated gold NPs showed higher antioxidative, anti-inflammatory, anti-proliferative, and anti-fibrotic activities in diethylnitrosamine-induced hepatocarcinogenesis in rats [79]. Graphene nanostars conjugated with a PAMAM-G5 dendrimer were prepared for the selective targeting and delivery of a plasmid expressing collagenase metalloproteinase 9 under the CD11b promoter into inflammatory macrophages in cirrhotic livers. The nanoformulations promoted the macrophage switch from inflammatory M1 to proregenerative M2 and reduced selectively and locally the presence of collagen fibers in fibrotic tracts [83].

Protein-based NPs currently show great potential as drug carriers for the treatment of liver fibrosis because of their biocompatibility and low immunogenicity (Table 1) [87]. Algandaby et al. reported that curcumin-loaded zein nanospheres showed high efficiency in attenuating the hepatic gene expression of collagen I, the tissue inhibitor of MMP2, and TGF-β, as well as downregulating MMP2 expression [84]. Moreover, compared with free berberine, berberine entrapped in glucose-modified albumin NPs more efficiently inhibited the growth of the human hepatic stellate cell line LX-2 and reduced liver fibrosis in vivo [85]. Human serum albumin-dexamethasone NPs were also fabricated to deliver dexamethasone to non-parenchymal hepatic cells, which play an important role in the pathogenesis of liver fibrosis. This treatment efficiently inhibited TNF-α production, hence the significant decrease in fibrosis relative to that in rats treated with free dexamethasone treatment [22]. Another study reported on the preparation of avidin-nucleic-acid-nano-assemblies (ANANAS), which are NPs based on polyavidin. These NPs were generated from a nucleic acid filament and avidin, a protein in egg whites. These NPs were designed to selectively deliver dexamethasone to the liver, particularly to the liver immunocompetent cells, and thereby improve the therapeutic efficacy by reducing interlobular collagen I deposition and MMP13 [86].

### 4.3. NPs as Drug Carriers with Targeting Ligands for the Treatment of Liver Fibrosis

Non-specific drug disposition limits the effective clinical use of traditional anti-fibrotic drugs. Targeting drug delivery to the fibrotic region can thus far be achieved using nanoformulations. As the sole hepatic VA storage cells with a crucial role in liver fibrosis, HSCs have been actively targeted by conjugating NPs with VA.

Liposomes loaded with drugs and HSC targeting components have been developed to target HSCs for the treatment of liver fibrosis (Table 2). In one study, VA-coupled liposomes were prepared to deliver imatinib. The hepatic accumulation of imatinib increased by about 13.5-fold, compared with imatinib treatment alone [88]. The nanoformulations not only inhibited the expression of phosphorylated PDGFR-β but also reduced the expression of profibrotic mediators such as hydroxyproline, TGF-β, and MMP2 with fewer adverse effects. In another study, VA-coupled liposomes were used to deliver valsartan, an angiotensin II receptor antagonist [89]. The nanoformulations increased the expression of hepatic Mas-receptor and PPAR-γ and potently normalized the level of fibrogenic mediators by improving the permeability and efficacy of valsartan.

**Figure 4:**
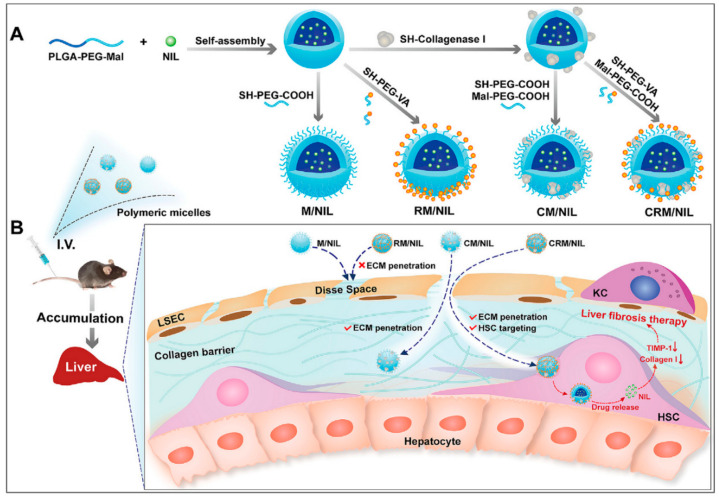
Extracellular matrix-penetrating polymeric micelles for liver fibrosis therapy. (**A**) Schematic illustration of the preparation of four different polymeric micelles. (**B**) Schematic illustration of the proposed destiny of the four different polymeric micelles in vivo. The CRM/NIL is able to penetrate the collagen barrier and target activated HSCs. Internalization of CRM/NIL allows the release of NIL, which reduces expression of the metallopeptidase inhibitor, TIMP-1, which in turn enhances collagen I degradation, thereby exerting therapeutic action against liver fibrosis. Reproduced with permission from [100]. Copyright Elsevier, 2020.

Polymer-based NPs have also been fabricated to target HSCs by coupling with VA for liver fibrosis therapy (Table 2). An article reported on the preparation of retinol and collagenase I co-decorated polymeric micelles (CRM) based on PLGA-b-poly(ethylene glycol)-maleimide (PLGA-PEG-Mal) to be used as HSC-targeting nanodrug delivery systems for liver fibrosis therapy [100]. In the current study, the decoration of collagenase I could facilitate the nanocarrier penetration of the fibrotic liver. Consistent with this finding, CRMs were found to efficiently degrade pericellular collagen I and exhibit excellent accumulation in the fibrotic liver and accurate targeting of activated HSCs in a mouse hepatic fibrosis model. CRM/NIL loaded with nilotinib (NIL), a second-generation tyrosine kinase inhibitor used for the treatment of liver fibrosis, showed excellent antifibrotic efficiency (Figure 4). In addition, polymeric micelles (PVMs) formed with PLGA-polyspermine-PEG-VA were used to target HSCs and deliver the chemical drug silibinin and genetic drug siCol1α1 to the liver fibrosis site [101]. The double-loaded polymer micelle more efficiently reduced collagen I and ameliorated liver fibrosis, compared with the PVMS loaded with either the chemical drug only or genetic drug only. Chondroitin sulfate micelles coupled with retinoic acid and doxorubicin (DOX) (DOX + RA–CS micelles) were selectively taken up in activated HSCs and hepatoma cells, but not in normal hepatocytes (LO2) [102]. DOX + RA–CS micelles preferentially accumulated in the Golgi apparatus, destroyed the Golgi structure, and ultimately downregulated collagen I production in vitro and exerted synergistic antifibrotic effects on CCl4-induced fibrotic rat models.

Apart from polymeric micelles, other polymer nanoformulations have also been constructed for the delivery of drugs, nucleic acid and other therapeutic moieties for the treatment of liver fibrosis (Table 2). In one study, retinol-conjugated polyetherimine NPs adsorbed plasma proteins, particularly retinol-binding protein 4 (RBP), forming a protein-coated complex [103]. The adsorbed RBP could direct the NPs into HSCs. After being loaded with antisense oligonucleotides, NPs effectively suppressed the expression of collagen I, consequently ameliorating hepatic fibrosis. Hassan et al. reported that chitosan NPs loaded with JQ1 (a small molecule that could abrogate the cytokine-induced activation of HSCs and reverse fibrotic response in animal models) and atorvastatin and further conjugated with retinol could target and prevent HSC activation [105].

In addition to VA, cyclic peptide pPB can particularly recognize PDGFRβ on the surface of HSCs (Table 2). A study used pPB-modified liposomes to deliver recombinant human tumor necrosis factor-related apoptosis-inducing ligand (rhTRAIL) to the HSC membrane, prolonging rhTRAIL circulation in vivo and alleviating fibrosis both in vitro and in vivo [90]. Similarly, the CXCR4 antagonist AMD3100 could target HSCs [91]. AMD3100-conjugated liposomes efficiently delivered therapeutic VEGF siRNAs to activate CXCR4-overexpressed HSCs both in vitro and in vivo. The nanoformulations downregulated the expression of VEGF, reduced the mean vessel density, and normalized the hepatic vascular structure in the livers of mice with CCl_4_-induced liver fibrosis. Moreover, AMD3100 encapsulated in liposomes also exhibited antifibrotic effects by suppressing the proliferation and activation of HSCs. Mannose 6-phosphate (M6P)/insulin-like growth factor-II receptor, overexpressed in HSCs, was also used as the targeting site. Conjugation of M6P-modified albumin to hesperidin-loaded liposomes improved the efficacy of chemical drugs and attenuated liver fibrosis [92].

Hepatic macrophages play important roles in the pathogenesis of liver fibrosis and act as target sites for the treatment of liver fibrosis (Table 2). Scavenger receptors expressed on liver endothelial cells and Kupffer cells have also been targeted using nanoformulations. For instance, phosphatidylserine (PS), which acts as a specific recognition signal for the phagocytosis of apoptotic cells, can target macrophages. Wang et al. showed that PS-modified lipid carriers containing curcumin (Cur–mNLCs) exhibited enhanced retention time, bioavailability, and delivery efficiency of payload, as well as reduced liver damage and fibrosis in vivo [99].

## 5. Nanomedicine in Liver Fibrosis Theranostics

**Figure 5:**
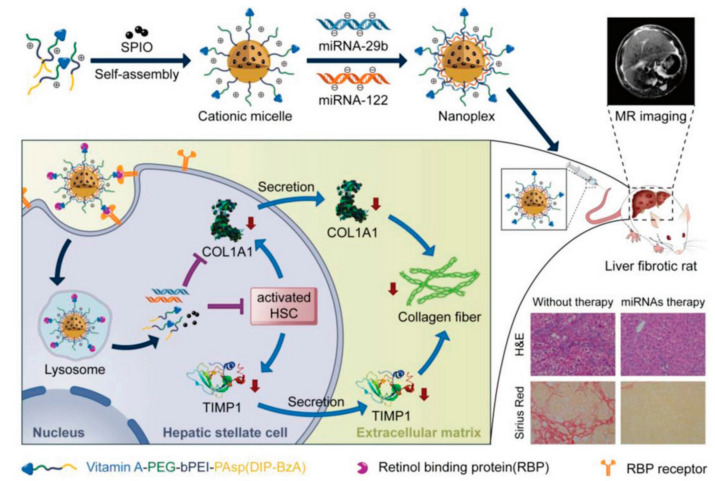
Vitamin A–decorated pH-sensitive and SPIO-loaded nanocomplex T-PBP@miRNA/SPIO (T-miRNA/S) for miRNA targeting delivery in the therapy of liver fibrosis. Expression of liver fibrosis-related genes for alleviating liver fibrosis were synergistically downregulated. The red arrows indicate the reduction of COL1A1, TIMP1, and collagen fiber. Abbreviations: COL1A1, collagen type I alpha 1 protein; TIMP1, tissue inhibitor of metalloproteinase 1; SPIO, superparamagnetic iron oxide. Reproduced with permission from [109]. Copyright John Wiley and Sons, 2019.

“Theranostics”, a portmanteau word of “therapeutics” and “diagnostics”, is achieved by incorporating diagnostic and therapeutic functions into a single nanoplatform. Theranostics has been proposed as a new and revolutionary therapeutic concept in several types of disease therapy, including that for liver fibrosis [107]. This strategy allows simultaneous diagnosis and treatment response by using personalized medicine with high accuracy and specificity. In one study, Hepatitis B core protein nanocages coated with RGD-targeting ligands (RGD–HBc/QR) exhibited selectivity to activated HSCs by targeting integrin α_v_β_3_ and efficiently inhibited the proliferation and activation of HSCs both in vitro and in vivo [108]. By encapsulating a quercetin–gadolinium complex and/or labeling it with NIR fluorescent probes (Cy5.5), the resulted nanoformulations (RGD–HBc/QGd) showed great potential as MRI contrast agents and NIR fluorescent agents for liver fibrosis diagnosis in vivo. Another study reported that relaxin-conjugated PEGylated superparamagnetic iron oxide NPs (RLX-SPIONs) showed specific binding and uptake in TGFβ-activated HSCs, as well as strongly attenuated cirrhosis and showed enhanced contrast in MRI [20]. Micelles coupled with inorganic materials were also developed for theranostics to treat liver fibrosis. A pH-sensitive and VA-conjugated copolymer cationic micelle that was coupled with a superparamagnetic iron oxide nanoparticle could transport miRNA-29b and miRNA-112 to HSCs in an MRI-visible manner. Synergistic antifibrotic therapeutic efficacy was achieved by downregulating the expression of fibrosis-related genes, including collagen Iα1, α-SMA, and a tissue inhibitor of MMP1 (Figure 5) [109].

## 6. Conclusions and Future Perspectives

This review summarizes the strategies being used to develop novel methods for the treatment of liver fibrosis on the basis of multifunctional NPs. The application of nanomedicine systems in the diagnosis and treatment of liver fibrosis is widely reported in the literature and continues to be a rapidly growing research field, with emphasis on active targeted drug delivery and theranostics. Numerous types of inorganic and organic NPs have been extensively investigated, including metal oxide NPs, metal NPs, liposomes, polymer NPs, dendrimers, protein NPs, and organic–inorganic hybrid NPs. Each type has its advantages disadvantages. Inorganic NPs are intrinsically robust with relatively low manufacturing costs, but their design flexibility and functionality are limited. Organic NPs possess broad design flexibility for integrating multiple functions into one platform but show structural instability and involve high manufacturing cost and fabrication complexity. Organic–inorganic hybrid NPs combine the advantages of organic NPs and inorganic NPs and thus are preferred in the development of theranostic platforms.

Although NPs have shown great potential for liver fibrosis therapy, they also exhibit hepatotoxicity [110,111,112,113,114]. The long-term hepatotoxicity of NPs should be carefully and systemically evaluated, particularly when they are used in patients with liver disease. Patients are more sensitive to NPs because of reduced self-protective mechanisms, decreased immune function, and lack of ability for self-repair. Studies have shown that exposure to NPs increases pathological damage [115,116,117]. Therefore, the health risks involved in the use of NPs for liver fibrosis therapy should be given significant attention.

Until now, lipid-based NPs were the only nanomedicine system that in the clinical stages of studies for the treatment of liver fibrosis. Lipid NPs delivering siRNA against heat shock protein 47 were developed to target HSCs and treat advanced liver fibrosis caused by NASH or hepatitis C virus infection. This nanomedicine system was in clinical phase 1b/2 and study results were safe and effective [118,119]. To improve the clinical applicability of nanomedicine systems in the future, the following directions should be considered: (1) Developing stimuli-responsive nanomedicine systems with high sensitivity, which can intelligently respond to endogenous or exogenous stimuli and release payload at targeting sites. (2) Employing an “all-in-one” strategy to develop smart nanomedicine systems that combine multiple functionalities, including targeted delivery, prolonged blood retention, enhanced tissue penetration and cellular internalization, responsiveness to stimuli, and disease progressive monitoring. (3) Systematic evaluation of long-term toxicity, immunogenicity, and pharmacokinetics of medicine systems. Notably, from the clinical use of the reported nanomedicine systems, only one example was performed. All obstacles should be overcome by designing and fabricating nanomedicine systems with appropriate components, surface chemistry, sizes, payloads, and specific target ligands before clinical translation.

## Figures and Tables

**Table 1:** NPs as drug carriers without targeting ligands in liver fibrosis treatment.

Nanoparticle Systems	NPs Formulation	Delivered Drug	Reference
Lipid-based NPs	RNA oligonucleotide-liposomal	MTL-CEBPA	[70]
	Cationic lipid NPs	small interfering RNA to the procollagen 1(I) gene	[20]
	Dexamethasone-liposomes	dexamethasone	[71]
Polymer-based NPs	Cationic nanohydrogel particles	anti-Col1α1 siRNA	[72]
	Ketal cross-linked cationic nanohydrogel	Cy5-labeled anti-col1α1 siRNA	[73]
	PLGA	phyllanthin	[74]
	PEG-PLGA or PEG-PLGA/PLGA NPs	sorafenib	[75]
	Eudragit(R) RS100 NPs (SMnps)	silymarin	[76]
Inorganic NPs	Rhodamine B (RhB)-mesoporous silica NPs (MSNs-RhB	salvianolic acid B	[77]
	Mesoporous silica NPs	siTnC	[78]
	PEG-AuNPs	hesperetin	[79]
	AuNPs and SiNPs	NO donors	[80]
	PtNPs	Curcumin	[81]
	Calcium phosphate NPs (CaP@BSA NPs)	TSG-6	[82]
	Graphene nanostars linked to PAMAM-GS dendrimer	Plasmid	[83]
Protein NPs	Zein nanospheres	Curcumin	[84]
	Glucose modify albumin NPs	Berberine	[85]
	Albumin NPs	Bexamethasone	[22]
	Polyavidin-based NPs	Dexamethasone	[86]

**Table 2:** NPs used in active targeting therapy of liver fibrosis.

Nanoparticle Systems	NPs Formulation	Delivered Drugs	Targeted Ligand	Targeted Structures	Reference
Lipid-based NPs	VA-liposomes	Imatinib	VA	HSC	[88]
	VA-liposomes	Valsartan	VA	HSC	[89]
	pPB-modified liposomes	Recombinant human TRAIL	pPB	HSC	[90]
	AMD3100-liposomes	Antiangiogenic siRNA	VEGF siRNAs	HSC	[91]
	M6P-bovine serum albumin (BSA)-conjugated-liposomes	Hesperidin	M6P	HSC	[92]
	VA-coupled liposomes	BMP4-siRNA	VA	HSC	[93]
	Cationic liposomes	Artificial microRNA	microRNA	CTGF	[94]
	Chol-PEG-VA-amphiphilic cationic hyperbranched lipoid (C15-PA)	SiCol I α1 and siTIMP-1	VA	HSC	[95]
	pPB-modified stable nucleic acid lipid	siRNAs against heat shock protein 47	pPB	HSC	[96]
	Galactosamine-phospholipid NPs	siRNA targets CTGF	galactosamine	hepatocytes and renal tubular epithelial cells	[97]
	SP94-LCPP (lipid/calcium/phosphate/protamine) nanoparticle	TRAIL plasmid DNA	hepato-cellular carcinoma (HCC)-targeting peptide (SP94)	hepatocellular carcinoma (HCC) cells	[98]
	Phosphatidylserine-modified nanostructured lipid NPs	Curcumin	phosphatidylserine	macrophage	[99]
Polymer-based NPs	VA-collagenase I-poly-(lactic-co-glycolic)-b-poly (ethylene glycol)-maleimide (PLGA-PEG-Mal) (named CRM) micelle	Nilotinib	VA	HSC	[100]
	Poly (lactide-co-glycolide)-polyspermine-poly (ethylene glycol)-vitamin A (PLGA-PSPE-PEG-VA) self-assembled into core-shell polymeric micelles (PVMs)	Silibinin genetic (siCol1 alpha 1) drugs	VA	HSC	[101]
	Retinoic acid-chondroitin sulfate micells	Doxorubicin	VA	HSC	[102]
	POEGMA-b-PVDM -VA micelle	NO	VA	HSC	[19]
	Retinol-conjugated polyetherimine (RcP) nanoparticle	Antisense oligonucleotide (ASO)	RcP	HSC	[103]
	PLGA NPs	R406	R406	Macrophages	[104]
	retinol-chitosan NPs	JQ1 and atorvastatin	VA	HSC	[105]
Inorganic NPs	pPB-MSNP	Erlotinib	pPB	PDGFRB	[106]

Abbreviations: VA, vitamin A; TRAIL, TNF-related apoptosis-inducing ligand; HSC, hepatic stellate cell; CTGF, connective tissue growth factor; MMP, matrix metalloproteinase; NO, nitric oxide; VEGF, vascular endothelial growth factor.
